# A comprehensive map of the evidence on the performance evaluation indicators of public hospitals: a scoping study and best fit framework synthesis

**DOI:** 10.1186/s12962-018-0166-z

**Published:** 2018-12-06

**Authors:** Kimia Pourmohammadi, Nahid Hatam, Payam Shojaei, Peivand Bastani

**Affiliations:** 1 Health Services Management, Student Research Committee, Shiraz University of Medical Sciences, Shiraz, Iran; 2 Health Services Management, Health Human Resources Research Center, School of Management and Medical Informatics, Shiraz University of Medical Sciences, Shiraz, Iran; 3 Department of Management, Shiraz University, Shiraz, Iran

**Keywords:** Hospital, Key performance indicators, Scoping review, Best Fit Framework Synthesis, Equity, Effectiveness, Efficiency

## Abstract

**Introduction:**

Key performance indicators are essential navigation tools for hospitals. They provide managers with valid information enabling them to identify institutional strengths and weaknesses and improve managerial performance. In this study, the synthesis of evidence relating to hospital performance indicators was carried out by means of a field review and the indicators were analyzed through the Best Fit Method.

**Methods:**

The five-step approach of Arksey and O’Malley was used as follows: selection of the research question; search for related studies; selection and refinement of the studies; synthesis and tabulation of key information; derivation of the related summary and report. Applying the Best Fit Framework Synthesis Method, the initial themes and subthemes were created and a model of public hospitals performance evaluation finally generated.

**Results:**

Forty-nine studies were considered eligible to form part of the synthesis. The final model included the efficiency/productivity, effectiveness and financial themes. The efficiency/productivity sub-themes incorporated human resources indicators, hospital beds, costs, operating room productivity, emergency rooms, ICU, radiology, labs, technology and equipment productivity. Other sub-themes relate to general indicators such as BOR, ALS, number of outpatients and hospitalized patients. Financial themes included profit, revenue, cash flow, cost, investment, assets, debt and liquidity. Concerning effectiveness, the indicators were categorized in terms of access (equity), safety, quality and responsiveness. The accountability indicators were classified into patient-centeredness, staff orientation, and social responsibility.

**Conclusion:**

Hospital performance management is a multi-dimensional issue, each dimension having its own significance. Based on the evidence, indicators are dependent on the evaluation model employed, the evaluation objective, and the views of executive managers and participants in the study. Selection of the most appropriate indicators is therefore key to a comprehensive performance evaluation system.

## Introduction

Health systems are today one of the largest sectors of the world’s economy and among the most important factors for community development and social welfare [1]. In the World Health Report, hospitals are identified as major health care providers and among the factors determining the equitable distribution of health care and promotion of the justice index in the health system. Furthermore, health systems realize their intermediate and final goals at all levels through enhanced hospital performance [2, 3]. Hospitals are the most essential and, at the same time, most costly part of the health system, so that in developed and developing countries 40% and 80% of the health sector expenses respectively are allocated to hospitals [4, 5]. In line with the rapid growth of expenses, environmental changes cause hospitals to face many political, economic, social and cultural changes over time. These changes include population ageing, advances in health technologies, development of information technology and telemedicine [6], all of which require rapid and active responses and measures. In this regard, appraisal of hospital performance indicators is an effective strategy for properly managing such changes. Continuous scrutiny of hospital procedures further prepares managers to proactively respond to these changes [7–9].

Key performance indicators (KPIs) are considered performance-based decision-making tools for policymakers and managers at national and local levels. These indicators provide valid information for managers, enabling them to identify their strengths and weaknesses and improve their managerial performance. Such information is also a good tool for the development and planning of promotional activities by organizations [10]. However, the paucity of evaluation and control systems in various dimensions such as resources, facilities, staff, goals and strategies means that there is no connection with the environment inside and outside the organization. This is considered to be one of the symptoms of organizations afflicted by disease, leading ultimately to their death [11].

Assessing clinical and economic performance indicators in hospitals helps policy-makers, managers and doctors to monitor performance and payment systems. It also promotes procedural transparency and individual accountability, resulting in better institutional performance [12]. Paying attention to hospital performance indicators is likewise conducive to achieving the hospital’s internal and external goals [13, 14], making effective and efficient use of available resources, improving service quality [15], and providing a clear perspective on hospital efficiency and effectiveness [16]. However, given the continuous changes in hospital performance, these indicators should be regularly reviewed on the basis of new evidence [17]. Identifying performance indicators not only helps to promote the responsiveness, efficiency and effectiveness of organizations as well as public trust in them, but also contributes to the planning and development of strategies to deal with complicated environmental changes [18]. The lack of an integrated and universally accepted framework for measuring health service performance has led various studies to examine different dimensions and indicators of hospital performance [19–21].

Some studies have employed procedures such as the Balanced Scorecard [19], Data Envelopment Analysis [22–24] and Pabon Lasso [24, 25] models, while others have concentrated on particular aspects of hospital performance. To evaluate and rank hospitals in New Zealand, Davis et al. focused on efficiency, effectiveness, and equity [26]. Pink et al. studied hospital performance in terms of financial performance, employing a review, panel and survey approach to assess the financial indicators of hospitals and reporting on them in terms of the five dimensions of financial sustainability, liquidity, capital, efficiency and human resources [27]. Gu and Itoh evaluated the views of 228 managers and 894 employees, and classified hospital performance indicators into 8 factors: survival and mortality rates, operational efficiency, patient/staff safety, financial effectiveness, quality of work life, staff development, patient-centered care, and patient/staff satisfaction [20]. Xenos et al. appraised the productivity and efficiency of Greek hospitals over a period of financial crisis [28]. Nikjoo et al. conducted a “mix method” study and selected key performance indicators (KPIs) for hospitals in the three areas of quality-effectiveness, financial-efficiency and access-equity [29]. In their study, Khalifa and Khalid identified 58 KPIs for hospitals, and categorized them into patient access, hospitalization utilization, outpatient utilization, operating room utilization, emergency utilization, general utilization, patient safety, infection control, documentation compatibility, and patient satisfaction [30]. There is no consensus regarding an effective approach to evaluating the performance of health services. In this regard, developing a combination of methods, frameworks and indicators for measuring hospital performance can provide a comprehensive perspective on hospital capabilities [2, 17, 23]. Evidence-based management focuses on integrating the findings of management research in the decision-making process of health system managers [31], preventing or minimizing overuse, underutilization and misuse of managerial activities. Such management further eliminates the gap between research and practice [32], making it possible to use the experience of other organizations and ameliorate the quality of decision making [32, 33].

Through a comprehensive review and summary of all studies on a given topic, knowledge synthesis interprets the results of those studies within a general evidential framework so as to provide policymakers and managers with assistance in planning and decision-making [34]. Given that summarizing and publishing research results is one of the main objectives of scoping reviews [35], the evidence about hospital performance indicators was synthesized in this study by means of a scoping review and the indicators were analyzed through the use of the Best Fit method.

## Method

In this study, a systematic scoping review carried out in 2018, the Arksey and O’Malley approach and the complementary recommendations of Livak were used to specify the performance indicators of public hospitals. The approach consists of five main stages and one optional stage as follows: selecting the research question, searching for related studies, selecting and refining the studies, synthesizing and tabulating key information, summarizing and reporting, and verifying and validating the results using the expert panel (optional) [35–37]. These stages are discussed as follows.

### Selecting the research question

The research question is “What effective performance indicators in public hospitals can be observed in the existing studies?”

### Data source and search

At this stage of the scoping review, the three main resources included electronic databases, reference lists of articles and a manual search of other resources, such as relevant key journals, networks, organizations and conferences. To ensure that the study was not reiterative, the studies registered at the Cochrane Library were the primary source, where no systematic reviews on the subject were found.

In order to identify the keywords, a pilot study was conducted by the information officer on the PubMed, Web of Science (ISI), Science Direct and SCOPUS databases separately. The pilot study showed that by using different keywords in each database, a higher percentage of related articles could be accessed. Table 1 presents the keywords suitable for each database. The main search on the intended databases was done in 2017, from July 26 to the end of December, without time limitations. In addition, so as to have access to new articles related to the subject, the researcher signed up to the databases and activated the alert option.

**Table 1:** Selected key words for study

Data base	Key words
Pub med-ISI Scopus	“General hospital “OR “public hospital” AND “PERFORMANCE” OR “ performance assessment” OR “ performance evaluation” OR “ performance monitoring” OR “ performance audit” OR “ performance survey” OR “ performance standard” OR “quality indicator” OR “quality assessment” OR “effectiveness indicator” OR “ efficiency indicator “ OR “ productivity indicator “ OR “safety indicator” OR “ profitability indicator “ OR “social responsibility” OR “general hospital effectiveness” OR “ general hospital efficiency” OR “ general hospital productivity” OR “general hospital safety” OR “ general hospital profitability” OR “ general hospital accountability” OR “ general hospital responsibility” OR “Effective Driven factors” OR “Performance Criteria” OR “Performance Criterion” OR “Decision making criteria” OR “Performance Index” OR “Performance Indicator” OR “Performance measurement” OR “Performance metric” OR “performance appraisal” OR “financial audit” OR “financial disclosure” OR “ financial performance”
Science direct	“General hospital” AND “Managements” OR “Commission on Professional, Hospital Activities” OR “Hospital Department” OR “Administration, Hospital” OR “Performances, Task” OR “Hospital Economics” OR “Hospital Financial” OR “Hospital Organization and Administration” OR “Association, American Hospital” OR “Healthcare Quality, Access, and Evaluation”

To increase sensitivity (i.e. to increase the selection of related articles), the researcher examined several databases, searched with relatively common terms, and used synonym words with the “OR” operator. In addition, in order to increase specificity (i.e. to reduce the selection of unrelated articles), synonyms were used with the “AND” operator. The search strategies are included in Appendix 1: Table 3. To ensure the comprehensiveness of the literature search, references to the selected and related articles were reviewed as well. Furthermore, a manual search was carried out on the resources of networks, organizations and conferences related to the topic, including unpublished studies of national or local organizations. In order to access unpublished information sources, experts in the field of hospital operation were contacted and access to the identified resources was obtained through personal visits or correspondence with the experts.

### Inclusion and exclusion criteria

The following criteria were used as a guide for searching and screening the articles. The inclusion criteria were English language studies, studies evaluating public hospital indicators, and original studies and reviews including systematic review, meta-analysis, meta-synthesis, scoping review, narrative review, rapid review, critical review, and integrative review. Studies on the indicators of health centers, the health system at the macro level, clinics and community health indicators, journals that did not have a precise review process, and articles such as book reviews, commentaries and opinion articles were adopted as the criteria for exclusion.

### Screening

The articles obtained from the search bases were individually reviewed by two people in three stages (title, abstract and full text). The final decision was made on the basis of agreement, which would require the comments of a third party if agreement was not reached. Screening was effected using the EndNote v.8 software. Given that quality assessment is not commonly performed in scoping reviews, the quality of the articles was not investigated in this research [38].

### Data extraction

According to the refined studies, the data were extracted in order to meet the research objectives and questions. To this end, a data extraction form was initially designed and tested on 10 randomly selected papers. Article authors, years, countries, types of study, study objectives, settings, and indices were extracted on this basis. At this stage, one of the authors extracted the data from the selected articles, and the second author examined the data. The form was designed and completed for each article in the Excel software.

### Data synthesis

**Fig. 1:**
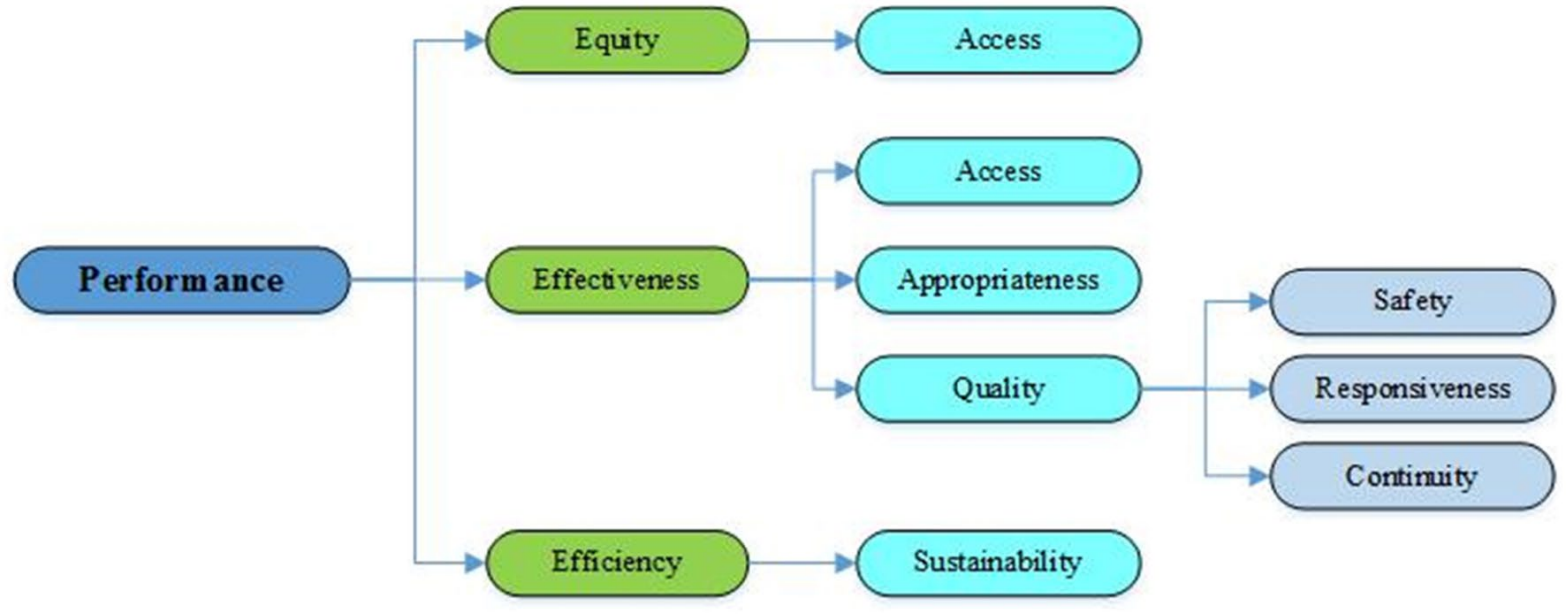
Initial themes reflecting the dimensions of public hospital performance evaluation, derived from literature [39]

The Best Fit Framework Synthesis Method was used to analyze the extracted data. In this way, the most suitable model related to the topic was selected, and the initial themes were created. The codes extracted from the articles were subsequently positioned in front of the themes [39, 40]. In the present study, the framework introduced by Australia was selected as the primary framework for the performance indicators of public hospitals, which were analyzed in terms of equity, effectiveness and efficiency [41]. According to this framework, the equity dimension includes the fair access indicators. Also the three dimensions of access, appropriateness and quality are used to assess service effectiveness. For the evaluation of quality, the model adopts the dimensions of safety, responsiveness and continuity of care. Finally, in order to assess efficiency, the sustainability of serviced was taken into account (Fig. 1).

Based on the Best Fit Method, the selected framework might change during the research and data collection, whereby a new conceptual framework could be generated [39]. Under this method, both deductive and inductive approaches were therefore used for data analysis [42] (Fig. 1).

Performance indicators were initially coded as semantic units. In the first stage, indicators related to the dimensions of the initial model were inserted deductively through explicit analysis. Specific words including equity, effectiveness, and efficiency were searched and their related indicators were identified and positioned through the closed coding method. In the second stage, indicators that were not included in the initial framework were classified inductively through the open coding method. For this purpose, the articles were studied one or several times for immersion. The indicators were then identified as semantic units through an implicit approach. In the following stage, the codes were grouped on the basis of semantic similarities. After that, the codes of each study were compared with those of other studies and ultimately classified as themes and sub-themes. Finally, the results of these two stages were put together and a new framework was created.

### Ethical considerations

Before using the open access studies, the journals or authors of the articles were contacted and their permission was obtained. In order to prevent bias, all stages of the study such as screening, data extraction and data analysis were carried out by two individuals.

## Results

**Fig. 2:**
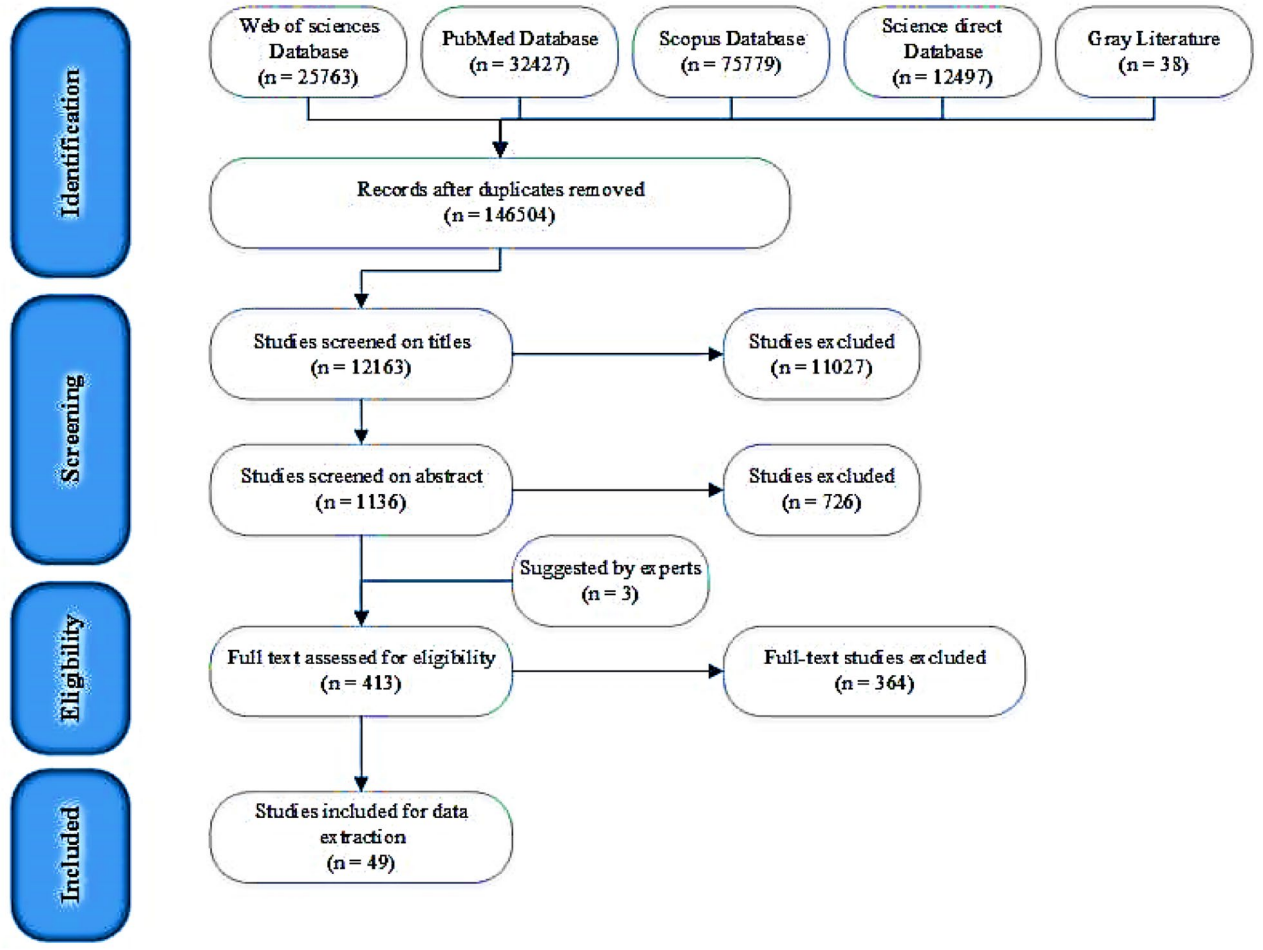
PRISMA Flow diagram for article selection

In the initial search, 146,504 English articles were found in scientific databases and by means of manual search, with duplicate and unrelated articles being removed, and 12,163 articles were reviewed. In the second stage, 1136 studies were reviewed based on their abstracts. As a result, 723 articles were excluded because they did not meet the inclusion criteria (413 ones were selected). Finally, after reviewing the full texts of the remaining articles, 49 ones were considered eligible to enter the study (Fig. 2). The features of these studies are summarized in Appendix 2: Table 4. Iran, USA and Brazil had 10, 8 and 5 articles respectively, Australia and Canada had 3, Britain, Turkey, Greece, and New Zealand had 2 articles, and Spain, Romania, Saudi Arabia and Japan had 1 paper; there was 1 article from the OECD countries and 1 from Nordic countries, and the other studies were reviews.

**Fig. 3:**
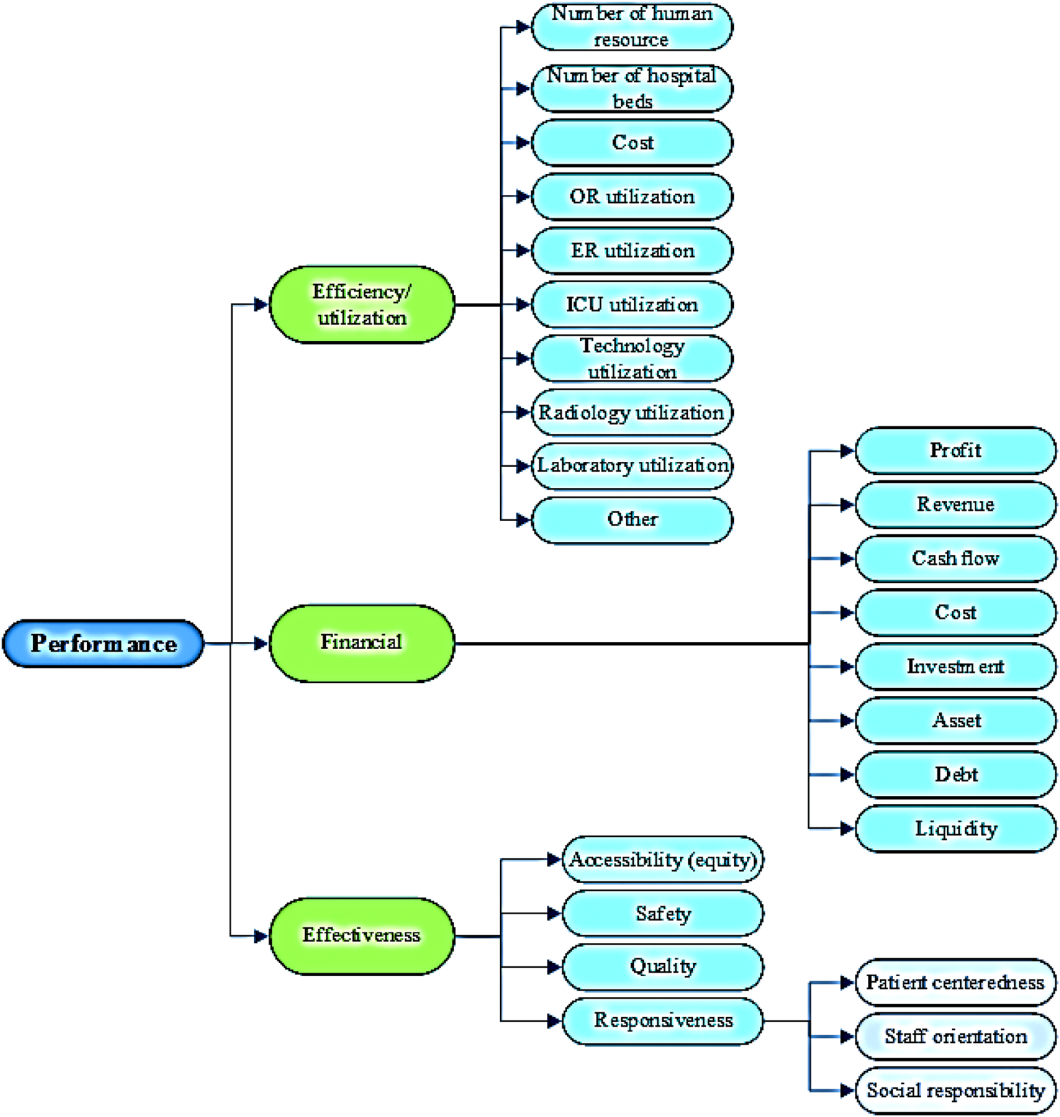
Final generated model of public hospitals performance evaluation

Based on the Best Fit Method, the final model included the efficiency/productivity themes, the effectiveness of the original model and the financial theme identified from the literature review (Fig. 3). The efficiency/productivity sub-themes included human resources indicators, hospital beds, costs, operating room productivity, emergency rooms, ICU, radiology, labs, technology and equipment productivity. Other sub-themes relate to general indicators such as bed occupancy rate, mean length of stay, number of outpatients and hospitalized patients. Financial themes were categorized into eight sub-themes including: profit, revenue, cash flow, cost, investment, asset, debt and liquidity. Concerning effectiveness, the indicators were further categorized into the four sub-themes of access (equity), safety, quality and responsiveness. The accountability indicators were classified into three categories: patient-centeredness, staff orientation, and social responsibility.

The indicators extracted from the studies are shown in Table 2 based on the final model. In this study, 173 indicators of public hospital performance evaluation were identified, most of which were in the effectiveness dimension (100 indicators). Regarding efficiency and financial dimensions, 41 and 32 indicators respectively were identified Best Fit Method.

**Table 2:** Taxonomy of hospital performance indicators

Theme	Sub-theme	Indicators	References
Efficiency/utilization (17 of the 49 included study)	Number of human resources	Number of physicians Number of nurses, Number of clinical personnel, Number of full-time equivalent interns/residents Number of administrative personnel, Number of nonclinical personnel, FTE/adjusted admissions	[20, 22–24, 45, 51, 52, 79, 80]
	Number of hospital beds	Percentage of specialized beds (%), Percentage of other beds The ratio of active beds to fixed beds	[25, 46, 70, 79, 80]
	Cost	Cost of medical/operating supplies Wage and salary payments to personnel engaged in patient/non-patient care Capital costs, i.e. building and land; Adjusted depreciation charges for fixed and movable equipment; Cost/adjusted admission Cost of inpatient services per patient day (cost per in-patient)	[22, 45, 47, 48, 50, 79]
	OR utilization	Number of OR cases booked Number of OR cases performed Number of OR cases cancelled Percentage of OR cancellations Percentage of surgical operations to surgery beds Day stay surgery rate	[19, 24, 26, 30]
	ER utilization	Total number of ER visits ER treatment time	[22, 30, 48]
	ICU utilization	Average ICU bed Occupancy rate Average ICU length of stay	[30, 48]
	Technology utilization	Use of electronic medical records, Rate of utilization of existing technology Number of high-tech services Number of medical supplies per bed Number of other operating supplies per bed Clinical integration (binary) Integrated data base	[20, 22]
	Radiology utilization	Total radiological procedures	[30]
	Laboratory utilization	Total lab investigations	[30]
	Other	Bed occupancy rate Average length of stay Bed turnover interval Monthly number of inpatients Monthly number of outpatients Average number of drugs per encounter	[19, 20, 22–25, 27, 29, 30, 46–48, 51, 53, 62, 66, 79, 80]
Financial (15 of the 49 included study)	Profit	Total marginal profit Medical benefit–cost–per FTE	[19, 81, 82]
	Revenue	Operating revenue per adjusted patient days Non-operating revenue Current ratio, n (%) = the ratio of net income (revenues/expenses) to total revenues Revenue per physician FTE	[19, 47, 51, 62]
	Cash flow	Cash to total debt	[47, 82, 83]
	Cost	Operating costs per adjusted patient days Unit Cost Performance, N (%) Cost of outpatient visits (primary vs. secondary cases) Cost of salaries and overtime (clinical vs. non-clinical staff) Emergency services expenses Personnel expenses Goods and services expenses Medicine expenses Average cost per day of hospitalization Pharmacy cost	[19, 62, 84]
	Investment	Return on investment	[27]
	Asset	Total asset turnover Tangible assets Return on assets	[27, 70, 82]
	Debt	Total debt/total assets Long-term debt to capitalization Debt ratio	[27, 47, 70]
	Liquidity	Current ratio Days revenue in net accounts receivable Days cash on hand Average payment period Replacement viability Acid test ratio Quick ratio Budget flow compared to approved budget	[27, 70]
Effectiveness (20 of the 49 included study)	Accessibility (equity)	Waiting time in emergency room Waiting time for initial clinical examination at the ER after arrival Waiting time for admission after arrival at the ER Waiting time for selective surgical treatment Patients leaving without being examined Outpatient appointment waiting lists Overall satisfaction rate of patient with nursing care Adherence rate to the patient satisfaction survey Outcome and satisfaction of complaints Communication/information Caring/compassion Ease of access Parking/food/other services Control of pain or other symptoms Expected results achieved Coordination of care Involvement of family and friends Respect for values and preferences Amenities Comprehensiveness Continuity	[19, 20, 29, 30, 52, 64, 65]
	Safety	Rate of nosocomial infections Rate of accidents Rate of complications Failure to rescue Incidents/near misses Accidents/adverse events Needle stick events Hospital-acquired infections Medical errors per sector Staff injury Staff needle puncture incidents Ventilator pneumonia Technical difficulty with procedure Medical equipment-related adverse event Patient falls Wrong surgery rate (wrong side, wrong body part, or wrong person) Hand hygiene compliance rate Postoperative respiratory failure Postoperative sepsis Prevalence of sentinel events	[19, 22, 30, 47, 48, 52, 53, 64, 66–68]
	Quality	Unplanned readmissions 30-day mortality Perioperative mortality Cancer patients successfully surviving surgery/chemotherapy/transplant The pure rate of hospital mortality, Success to hospitals in obtaining certificates of management quality Appropriateness of care (caesarean section rate) Surgery postponed or canceled Management team participation in Quality Improvement (QI) programs (Board activity in QI, CEO participation in QI activities, Board monitoring of QI, Clinic audit meetings held, Perceived barriers to QI) Diffusion of QI across hospital units Proportion of FTEs on QI teams Proportion of physicians on QI teams Management of hospital waste Number of guidelines developed Proportion of physicians using guidelines Staffing level and training hours (for staff with direct patient contact) A patient safety committee A system for reviewing patient deaths Policies for handling dangerous chemicals A credentialing committee Quality of life used to assess organizational performance Technical quality of care Appearance of facilities	[19, 20, 26, 29, 47, 48, 52, 53, 64, 65, 68, 69]
	Responsiveness	*Patient centeredness* Patient feedback management Pain control Satisfaction from personnel Explanation of procedures treatment and discharge information Satisfaction from hospital environment *Staff orientation* Staff burnout Staff absenteeism Staff working overtime Satisfaction from working environment Clearly defined responsibilities in staff average payment Diversity Working hours Frequency of night duty/shift Occupied position Average experience in current dept. Staff safety Number of work-related injuries Paid leave Number of staff per bed Continuous education for health professionals Number training hours on total number of working hours Training budget on total budget dedicated to staff Vacancy *Social responsibility* Leadership and inner processes which include the areas of mission and vision, policies and procedures, ethical codes, regulations and procedures Marketing that refers to suppliers and contractors, supply chain, consumer rights, responsibilities and liability management services including responsible purchasing Workplace environment which contains staff safety and health issues An environment which includes issues of sustainable development, pollution, waste management, energy saving and green purchasing management Community that states the local community, academic community in partnership with social institutions, partnership with non-governmental organizations (NGOs), volunteer participation supporting activities of employee and charitable support Provider mix reflective of community(ies) served Governing board and management staff reflective of community(ies) served Community Benefit Care provided in public programs (e.g., Medicaid) Numbers served in free clinical service programs (e.g., blood pressure screening, immunizations)	[19, 20, 29, 30, 47, 48, 52, 53, 64–67, 69, 70]

## Discussion

As demonstrated by this and other studies, there exist various objectives, fields of inquiry and methodological approaches when it comes to evaluating hospital performance; with each study having its specific objective and approach (Appendix 2: Table 4). In any study, performance evaluation frameworks and indicators are selected and evaluated according to the objective of the study. The resulting differences may be due in part to national policies and plans or to technical differences in the health systems of countries [26]. However, the experience of different countries in selecting and using the indicators can be useful to policymakers, health managers and researchers in other countries [43]. The present study seeks to present the indicators used to evaluate hospital performance in the form of a comprehensive package. The indicators concerned have been classified under three main headings (efficiency/utilization, finance and effectiveness), as discussed below.

Analysis of the selected studies shows that the model adopted in this study differs from the original model (Figs. 1, 3). In the original (Fig. 1), equity (access) was considered a major dimension of hospital performance as well as one of the subsets of effectiveness. Given that most studies assigned indicators of equity in access to the effectiveness dimension, and that this dimension was in practice often used in macro-decisions of the Ministry and was less likely to come within the scope of the authority of hospital managers [1–7], access (equity) in the proposed model was considered one of the subsets of the effectiveness of hospital services, along with other indicators such as safety, quality and responsiveness. In the proposed model, safety and responsiveness were included among the main subsets of effectiveness in view of their importance in hospitals.

Another dimension of the original model was efficiency, which was developed in the proposed model in view of the variety and diversity of the indicators used in previous studies. The indicators of efficiency were organized into ten sub-categories, most of which emphasized utilization of resources and equipment in different parts of the hospital, such as the operating room (OR), emergency room (ER), ICU and laboratory.

The results of this review showed that financial issues were of great importance in hospital performance evaluation studies. Limited financial resources and increased hospital expenses could explain why directors and researchers tend to focus on financial areas. However, new models and frameworks in the field of performance evaluation emphasize the multidimensional aspects of hospital performance and underline that other dimensions, in addition to finance, need to be taken into account [8]. In the proposed model, the effectiveness dimension, including the aspects of quality, safety, access, suitability and responsiveness, also has its place. Service effectiveness and improvement are not only factors of customer satisfaction (including patients, staff and the wider community) but also help to reduce costs and increase hospital income. In what follows, we discuss the dimensions of the proposed model in more detail.

### Efficiency/utilization

One of the challenges faced by health managers throughout the world is hospital efficiency [26] given that hospitals represent a large proportion of national health expenditures. In 2012, hospitals accounted for about 30% of total health expenditures in the OECD countries and 37% in the EU countries [28]. In their study, Lotfi et al. described hospitals as “organizations with inefficient resource management, low profitability, and low-quality services” (especially in developing countries). They stated that this poor management entailed a waste of resources and was a barrier to the efficiency of hospitals. Efficiency is therefore one of the most important factors in performance management systems in health-care organizations [23, 24, 44].

In the present study, several indicators were employed to evaluate efficiency as an major dimension of hospital performance. In the framework provided by WHO, efficiency is one of the six main dimensions of hospital performance evaluation [17]. Based on the findings, 17 studies used efficiency indicators in evaluating the performance of hospitals [20, 22–24, 26–29, 45–53]. These indicators were categorized under the sub-themes of human resources, hospital beds, costs, operating room productivity, emergency rooms, ICU, radiology, laboratory, technology and facilities productivity. Some of the most important indicators of efficiency are the number of human resources, bed occupancy rate, length of stay, utilization rate of the existing technologies, and the rate of drug prescription [47, 48].

*Human resources*, are considered important aspects of hospital efficiency evaluation [46, 54]. For instance, the number of hospital staff per bed is a key indicator in evaluating hospital performance and efficiency. The lower this ratio, the more productive and efficient the hospital will be [50]. The quality of care is another major indicator that must be taken into consideration. Additionally, a very low rate of bed occupancy, which represents the rate of *hospital bed* use, indicates a low level of hospital efficiency, which is highly correlated with the patients’ length of stay and bed turnover [46].

Another important issue in evaluating hospitals efficiency is *cost*. In their study, Pink et al. aimed to select key financial indicators for Ontario hospitals, and considered efficiency to be one of the five main dimensions of hospital financial performance. They measured efficiency indicators in terms of the ability to provide services at the level of predicted costs and to minimize management costs. They further selected the cost performance index of departments (units) and the percentage of corporate services as measures for evaluating hospital efficiency [27].

*Operating rooms* (ORs) are among the most vital and expensive parts of hospitals since 60% to 80% of hospital admissions involve surgical interventions. This sector accounts for more than 40% of the total hospital costs and a large proportion of hospital income [55, 56]. Utilization of OR affects the outcomes of surgical patients in hospitals so that even a small problem in the OR process can impact on the overall quality and performance of the hospital. Inefficiency of OR lead to delays in service delivery to patients, which can result in dissatisfaction on the part of patients and health care providers [55]. Hence, with the increase in financial pressures, most hospitals are looking for ways to enhance their income and reduce avoidable costs through the evaluation of OR processes. Given the impact of OR performance on hospital productivity, assets and personnel, many hospitals are devoting substantial resources to improving efficiency in this regard [55, 56].

*Emergency departments* play a major role in hospital performance since they deal with the most numerous, diverse, troubled and sensitive groups of patients, requiring prompt care and service [57, 58]. The number of patients treated and the duration of treatment in the emergency department were identified in the present research as indicators of efficiency and utilization of emergency departments. In the study by Kang et al., the most important emergency performance criteria were the timing of the various stages of emergency processes and the number of patients (admitted, in the waiting queue, and cancelled appointments) [58]. Horwitz et al. introduced the waiting time and length of visit as important indicators of the efficiency, timeliness, safety and patient-centeredness of emergency care [59].

The DEA and Pabon Lasso approaches are two of the most widely used methods for evaluating hospital efficiency. Using hospital indicators, both methods consider hospital inputs and outputs to measure efficiency. DEA is a linear programming approach that examines the relationship between hospital inputs and outputs, comparing them with the ideal (optimum) process [9, 23, 28, 45, 48]. Although there are limitations in linking inputs to outputs or health care outcomes (such as the lack of activity-based costs), there are also opportunities in measuring efficiency via the optimal use of available and accessible technologies, productivity rate, staff ratios and financial management [17].

### Finances

One of the common dimensions of performance evaluation is the financial aspect [20, 60]. In this regard, hospital financial models are unique in terms of their design and application and are affected by a hospital’s mission, goals, financing and accounting methods; the needs of population covered; the form of insurance reimbursement and the type of ownership. Hospital managers can overcome the hospital’s economic problems, make the right decisions, clarify the unit cost of services and create a competitive situation to provide goods and services applying a suitable financial evaluation model [61].

The results of this study indicated that 15 studies used financial indicators in evaluating hospital performance [19, 22, 27, 29, 30, 45, 50, 62, 63]. Based on the literature review, the different indicators used to evaluate financial performance are categorized into 8 sub-themes including *Profit*: total marginal profit, medical benefit–cost–per FTE); *Revenue*: operating revenue per adjusted patient days, non-operating revenue, current ratio, revenue per physician FTE; *Cash flow*: cash to total debt; *Cost*: operating costs per adjusted patient days, unit cost performance, cost of outpatient visits, cost of salaries and overtime, emergency services expenses, personnel expenses, goods and services expenses, medicine expenses, average cost per day of hospitalization, pharmacy costs; *Investment*: return on investment; *Asset*: total asset turnover, tangible assets, return on assets; *Debt:* total debt/total assets, long-term debt to capitalization, debt ratio; and *Liquidity*: current ratio, days revenue in net accounts receivable, days cash in hand, average payment period, replacement viability, acid test ratio, quick ratio, budget flow compared to approved budget) [61–66].

Classification of financial indicators focuses on the financial status of a hospital. Since the evaluation of each dimension of financial performance by itself may lead to a wrong decisions and plans, it is necessary to review them simultaneously. For instance, the evaluation of profitability indicators demonstrate the financial gain of a hospital, but liquidity indicators may suggest the inability of the hospital to pay off debts (bills) [27, 61]. Indicators of net profit or loss and operating profit or loss only represent and analyze the balance between income and expenses [60].

Along with what has been discussed and per the current environment in Iran, the poor economic condition and political sanctions have a detrimental influence on Iranian hospital financial performance and cause financial distress. Early detection of this condition by hospital manager is critically important. Many studies mentioned that the most effective and operational index in this regard is the cost/revenue ratio in public governmental hospitals [19, 59, 84].

### Effectiveness

Failure to provide effective health services reduces the quality of life, increases the burden of disease and disability and finally prevents the promotion of productivity in other economic, social and political areas [49]. The need to provide effective services has therefore always been a major issue. Performance measurement is a tool for evaluating the effectiveness of any organizational activity [47]. Thus the studies of Braithwaite et al. on eleven identified frameworks found that the effectiveness dimension had the most frequent replication in the performance evaluation frameworks [43].

Based on data extracted from the literature, 20 studies used indicators related to the effectiveness of hospital services [19, 20, 22, 26, 29, 30, 47, 48, 52, 53, 60, 63–71], categorized in the four sub-themes of access (equity), safety, quality and responsiveness. Although hospitals have tended to concentrate on improving efficiency (until the 1990s), recent efforts have addressed the issues of safety, quality, responsiveness and equity [26, 71].

First of all, the effectiveness of health services depends on the fair access of people to health services [26]. *Access* to medical care is a relatively complex multidimensional issue. From the perspective of a behavioral model, access includes six dimensions: potential access, achieved access, fair access, unfair access, efficient access and effective access [72]. In the Australian health performance framework, access to services was mentioned as part of the hospital performance evaluation. For instance, waiting times for elective surgeries and waiting times in emergency rooms were indicators of access to hospital services. The waiting time for surgery is indicative of the timeliness of the provision of services based on need [73]. In the study of Khalifa et al., patient access indicators included the number of referred patients, admitted patients and those waiting in line for admission [30]. Nerenz et al. considered easy access and waiting time as factors affecting patient satisfaction [60]. Ioan et al. also considered access and equity as aspects of hospital responsiveness [63]. In their study, Davis et al. used ethnic, social, and economic diversities to evaluate equity [26].

Another factor influencing the effectiveness of hospital activities is the ***quality*** of the services provided [74]. Quality of care refers to the clinical content of the care provided for a specific group of patients. However, it also includes certain quality indicators such as hospital infection or satisfaction of all patients admitted to the hospital [60]. Quality influences the effectiveness of activities as well as financial performance through its impact on profitability, cost, customer loyalty, and customer attraction [75]. Thus, quality is a key determinant of market share, return on investment, and cost reduction [76]. So, the need for evidence-based decision-making, measurable improvement, and useful information for comparison has led to an increasing emphasis on quality assessment in the health system [48]. However, the existence of unrestricted indicators related to the quality of services has rendered this dimension of performance evaluation heterogeneous. In the presented frameworks, quality indicators were categorized in different ways. For example, in the Donabedian model, quality was represented by the three concepts of structure, process, and output [60]. The SERVQUAL model also classified service quality into five categories: tangibles, reliability, accountability, service assurance, and empathy [77, 78]. Thus, the vital position of performance quality for all health beneficiaries (specialists, policymakers, service providers and service recipients) has led several studies to focus on the quality of hospital services and various indicators to be used in relation to their objectives.

Another factor influencing the effectiveness of hospital activities is the *safety* of the services provided. Although safety is one of the basic principles and elements of quality, it has recently been studied separately in certain cases [68]. Patient safety is focused on treatment effectiveness, and its indicators directly reflect treatment effectiveness [30, 68]. In various studies, safety has been considered a dimension of hospital performance evaluation, including the safety of patients, personnel and environment [17, 63]. The framework presented in the study by Veillard et al. highlighted the central role of safety in the governance of health systems and hospital management. Patient safety includes issues such as the development and use of standard guidelines, quality monitoring, issuance of prescriptions and drug delivery, infection control mechanisms, continuing care and professional qualifications [17]. McLoughlin et al. selected 21 indicators for countries and classified them into five categories: hospital infections, operation and postoperative complications, sentinel events, midwifery, and other care-related incidents [68].

*Responsiveness* indicators, based on patient feedback, are of great importance in evaluating hospital performance. In certain studies, responsiveness has been regarded as a separate dimension of hospital performance [30, 48]. Based on the analyses conducted in this study, responsiveness encompasses three fields:

*Patient centeredness* is defined in terms of patient feedback management, patient satisfaction, personnel and hospital environment, patient autonomy (meaning explanation of procedures and informed selection of treatment by the patient), dignity of patients, confidentiality, prompt attention, basic amenities and a social support network;

*Staff orientation* covering staff burnout, absenteeism, overtime worked, satisfaction with working environment, clearly defined responsibilities, average remuneration, diversity, working hours, frequency of night duty/shift work, position occupied, average experience in current department, personnel safety, number of work-related injuries, paid leave, number of staff per bed, continuous education for health professionals, number of training hours against total number of working hours, training budget against total budget dedicated to staff and vacancy;

*Social responsibility* is described by leadership and inner processes (including mission and vision), policies and procedures, ethical codes, regulations and procedures, marketing in terms of suppliers and contractors, supply chain, consumer rights, responsibilities and liability management services (including responsible purchasing) and the workplace environment (including staff safety and health and issues of sustainable development, pollution and waste) [75–78].

This approach is in accordance with Simou et al. who classified responsiveness indicators under the two categories of patient centeredness and staff orientation [48]. These various indicators show the wide compass of this dimension and the importance of this aspect in hospital performance evaluation.

The foregoing indicators in the field of hospital management are extracted from the entire range of existing literature and derived from various countries with a diversity of policies, cultures and rules. It is claimed that careful and comprehensive consideration and categorization of these indicators yield a conceptual framework that can be used as a basic theory and model synthesis worldwide, while remaining subject to adjustment and customization according to each country`s culture, rules and policies and the structure of the health system concerned.

## Conclusion

Hospital performance management is a multi-dimensional issue, with each dimension having its own significance. One-dimensional performance evaluation can lead to incorrect policy-making and decisions. On the other hand, several indicators of diversity in the literature highlight the scope and complexity of hospital performance. Based on the evidence, indicators are dependent on the evaluation model employed, the evaluation objective and the views of executive managers and participants in the study. It follows that a comprehensive and complete performance evaluation system is conditional upon the selection of the most appropriate indicators as a first step.

### Practical implications

#### Background

Key performance indicators (KPIs) are considered essential decision-making tools for policymakers and managers at national and local hospitals.

#### Purpose

Developing a comprehensive framework to provide the indicators used to evaluate hospital performance.

#### Methodology

The synthesis of evidence on hospital performance indicators was carried out through a scoping review and the indicators were analyzed using the Best Fit Method.

#### Results

Based on the Best Fit Method, the final model included the topics of efficiency/productivity, the effectiveness of the original model and the financial aspects as identified from the literature review.

#### Conclusion

Through a comprehensive review and summarization of all studies related to the same research question, knowledge synthesis interprets the results of those studies within a general framework of evidence, ultimately helping policymakers and managers with planning and decision making.

#### Practical implications

Hospital performance management is a multi-dimensional issue, with each dimension having its own significance. One-dimensional performance evaluation leads to incorrect policy making and decisions. On the other hand, several indicators of diversity in the literature highlight the scope and complexity of hospital performance. Based on the evidence, indicators are dependent on the evaluation model employed, the evaluation objective, the views of executive managers, and the study participants. It follows that a comprehensive and complete performance evaluation system is conditional upon the selection of the most appropriate indicators as a first step.

## Appendix 1

See Table 3.

**Table 3:** The search strategy

	Keyword	Location of the keyword	OR/AND	Keyword	Location of the keyword
1.	“Hospital index”	Title/abstracts	–	–	–
2.	“Hospital index”	Title/abstract/keywords	–	–	–
3.	“Hospital indicator”	Title/abstracts	–	–	–
4.	“Hospital metrics”	Title/abstracts	–	–	–
5.	“Hospital audit”	Title/abstracts	–	–	–
6.	“Performance measurement”	Title/abstracts	AND	Hospital	–
7.	“Performance evaluation”	Title/abstracts	AND	Hospital	–
8.	“Performance appraisal”	Title/abstracts	AND	Hospital	–
9.	Financial audit	Title/abstracts	AND	Hospital	–
10.	“Financial disclosure”	Title/abstracts	AND	Hospital	–
11.	Hospital administration	Title/abstracts	–	–	–
12.	“Hospital appraisal”	Title/abstracts	OR	Hospital evaluation	Title/abstracts
13.	“Financing”	Title/abstracts	AND	Hospital	Title/abstracts
14.	“Performance indicator”	Title/abstracts	AND	Hospital	Title/abstracts
15.	“Effective driven factors”	Title/abstracts	AND	Hospital/performance	Title/abstracts
16.	“Effective index”,	Title/abstracts	AND	Hospital/performance	Title/abstracts
17.	“Effective indicator”	Title/abstracts	AND	Hospital/performance	Title/abstracts

## Appendix 2

See Table 4.

**Table 4:** Summary of characteristics of included studies

	First author (year)	Country	Study design	Aim of study	Setting
1.	Vera Antonia Büchner (2000–2011)	Germany	Quantitative, cross-sectional	Investigates potential changes in hospital performance after health system entry	833 hospitals
2.	Marcelo Cristiano de Azevedo, (2012)	Sao Paulo, Brazil	Quantitative, cross-sectional	If size, administrative level, legal status, type of unit and educational activity influence the hospital network performance	533 hospitals
3.	Duygu Kirgin Toprak (2010)	Turkey	Quantitative, cross-sectional	Explore the effect of having ISO 9000 certification on the performance of public hospitals	146 hospitals
4.	Neidamar Pedrini Arias Fugaça (2014–2015)	Brazil	Qualitative field study	To develop a proposal for a nursing panel of indicators	200 medical records of patients
5.	Jack Zwanziger (1990–1999)	USA	Quantitative, cross-sectional	Study the impact of safety net activities on total profit margins and operating expenditures	16,680 Hospital*year observations.
6.	Joel Kupersmith (1989–2004)		Literature review	To compare the quality of care in teaching hospitals with that in nonteaching hospitals	
7.	Josue Patien Epane (2007–2014)	USA	Quantitative, longitudinal	Explores the impact of hospitalists staffing intensity on hospitals_ financial performance	4354 hospitals per year
8.	Asgar Aghaei Hashjin (2002–2008)	Iran	Mixed method longitudinal	To describe the development and process of implementation of the HPMP, and to explore the impact On hospital performance	696 hospitals
9.	Natalie Taylor (2000–2014)	Australia	Systematic review	Undertake a systematic review of qualitative literature to identify methods used to identify high performing hospitals, the factors associated with high performers, and practical strategies for improvement	19 studies
10.	P. Xenos (2009–2012)	Greece	Cross-sectional	To examine the dynamics of efficiency and productivity in Greek public hospitals	117 public hospital
11.	Amy K. Rosen (2005–2006)	USA	Quantity	Find the relationship between safety climate and safety outcomes in hospitals	30 hospital
12.	Denise Fornazari de Oliveira (2003)	Brazil	Quantitative and qualitative cross-sectional	Obtaining subsidies for standardization of a quality assessment program	1129 patients
13.	Anam Parand (1983–2010)	UK	Systematic review	To review the empirical literature to identify the activities, time spent and engagement of hospital managers in quality of care	19 articles
14.	Renu Agarwal (2016)	Australia	Mixed cross-sectional	To investigate the quality of management practices of public hospitals in Australian healthcare system	Benchmark in 7 countries (Sweden, USA, UK, Germany, France, Italy and Canada)
15.	Abhijit Basu (2006–2007)	UK	Quantity, cross-sectional	Analysis the data regarding the different clinical quality performance indicators mentioned in The Intelligent Board (2006) and to determine whether the results could be reliably used to interpret a hospital’s performance	One hospital
16.	Peter Davis (2001–2009)	New Zealand	Quantitative, Cross-national	Focus on evaluating hospital performance, using the New Zealand public hospital as a pragmatic application. Present descriptive results for the efficiency and effectiveness measures. In each case values for hospitals are ranked. Using the coefficient of variation	35 hospitals
17.	Xiuzhu Gu (2016)	Japan	Quantitative Pilot study on hospital management pis	To capture factors behind professional views of indicator usefulness as a common structure for assessing healthcare performance and their important characteristics to design limited key performance indicators (pis) for holistic hospital management	228 manager and 894 staff responses
18.	Mohammad Mehrtak (2014)	Iran	Quantitative, cross-sectional and retrospective	To measure the efficiency of hospitals Employing two distinct methods	18 general hospital
19.	George H. Pink (2000–2005)	Canada	Mixed Descriptive statistics, histograms and scatterplots were used to verify programming accuracy	This article describes the method used to select key financial indicators for inclusion in *hospital report*. And describe the literature, panel and survey approach that was used, and we present the results for five years of recent data for Ontario hospitals	12 hos
20.	Vivian G (2004)	USA	Quantitative cross-sectional	Analysis to assess the trade-offs between quality and efficiency in U.S. hospitals	1,377 urban hospital
21.	Effie Simou (1980–2010)	Greece	Mixed A literature review and a consensus panel	Describes the development of a preliminary set of quality indicators, which were used in the “health monitoring indicators system project, with the purpose of assessment the quality of all the aspects relevant to public hospital healthcare workforce and services provided	
22.	Dan Culica (2003–2005)	USA	Quantitative	To estimate the association between dimensions of board infrastructure and dynamics to their hospital financial outcomes. In addition, the extent to which financial performance was influenced by the board activities and infrastructure was explored	75% of hospitals
23.	Bahram Nabilou (2009–2014)	Iran	Quantity, cross-sectional	Determine the total factor productivity and its components over the period under the study.	17 hospital
24.	Nermin Ozgulbas (2004)	Turkey	Quantitative	Presents an application of the data mining method to determine the financial profiles Of the public hospitals	645 public hospitals
25.	John E. Schneider (1997–2004)	USA		Focuses on one important economic question: does the presence of specialty hospitals in a market affect general hospitals’ financial performance?	
26.	Kristin L. Reiter (1999 and 2006)	Canada	Quantity, cross-sectional		92 hospital
27.	Jeffrey A. Alexander (2006)	USA	Quantity	The objective of this study was to examine the association between the scope and intensity of Quality improvement (QI) implementation in hospitals and organizational performance	1784 community hospitals
28.	Hamed Rahimi (2017)	Iran	Mixed method	Organize suitable key performance indicators for hospitals’ performance evaluation based on the balanced scorecard	17 expert panel
29.	Goshtasebi, A (2005–2006)	Iran	Cross-sectional	The Pabon Lasso model was applied to assess the performance of six State-run hospitals	6 hospital
30.	Jack Needleman, Ellen T. Kurtzman, Kenneth W. Kizer	-	Review	Reviews efforts and issues involved in identifying a set of nursing-sensitive performance measures	-
31.	Carmen Silvia Gabriel (2009 to January 2010)	Sao Paulo, Brazil	Quantitative	To identify performance indicators adopted by the Nursing Service of a public hospital and to analyze the opinions of the nurses regarding the use of these indicators to evaluate the quality of the nursing care	
32.	Thomas N. Chirikos (1982–1993)	USA	Quantitative	To compare the results of scoring hospital efficiency by means of two new types of frontier models, Data Envelopment Analysis (DEA) and stochastic frontier regression (SFR)	186 hospital
33.	Mahmoud Keyvanara (2013)	Iran	Cross-sectional	Suggesting a new paradigm in hospital governance, the aim of this study was to measure the social responsibility in hospitals	21 hospital
34.	Raana Gholamzadeh Nikjoo (2010)	Iran	Quantitative-qualitative study Literature review & AHP	The aim of the present study is to identify and to select key hospitals performance indicators.	8 expert panel
35.	Vivienne McLaughlin (2005)	OECD countries	Qualitative and quantitative structured reviewand panel	Quality Indicator Project, which aimed at developing an initial set of patient safety indicators	
36.	John ovret veit (2001)	Nordic countries	Work shop-literature review	Provide an overview for non-specialist of the different quality evaluation and indicator scheme for inspection and improvement	
37.	Phil Hider (2001–2009)	New Zealand	Quantity	The risk adjustment accounted for age, gender, ethnicity, rurality, deprivation and comorbidities and was undertaken with SAS^20^ software	91 hospital
38.	Farhad Lotfi (2007–2011)	Iran	Quantity Cross-sectional	To obtain an overview of hospitals’ performance status by applying, hospital performance ratios, Data Envelopment Analysis (DEA), stochastic frontier analysis (SFA), Pabon lasso to compare similarities and differences between these methods and suggest the most comprehensive and practical method of appraisal for managers and policy makers. By Windeap 2.1 software	16 hospital
39.	Conference (2014)	Ministry of health & HIMSS Middle East			
40.	Mohamed Khalifa (2014)	*Saudi Arabia*	Qualitative	Decided to develop and utilize a group of strategic kpis to monitor, measure and improve the performance of the hospital including different departments and services	All professionals who belong to this group of executive and leading function
41.	Peivand Bastani (2008)	Iran	Quantity cross-sectional	This study was designed to present and compare Iranian hospitals` performance applying ratio analysis technique.	139 hospital
42.	Barliba loan (2008)	Romania	Quantitative cross-sectional	Focus on a specific stage of the research, namely: testing the managerial relevance of kpis, as a main element for validating applicability of the suggested model	5 hospital
43.	Report Canadian institute for health information (2013)	Canada	Report	To inform the development of the hospital performance framework	–
44.	David R. (2001)	–	–	Briefly summarize the history of performance measures for hospitals, health plans, and health care systems	–
45.	Olimpio J. (1996)	Sao Paolo, Brazil			
46.	Jérémy Veillard (2003)	Spain	Mixed	Twelve responses came from the 11 countries. One questionnaire was sent to each one of the countries. Surveys were filled in either by individuals or by large multi-professional working groups	200 hospital
47.	Beth Engel Brecht (2000)	–	–	It provides a framework towards a well-managed District Hospital	–
48.	Jenny Hargreaves (2008–2009)	Australia			
49.	Khayat Moghadam Saeed (2014)	Iran	Descriptive-analyzed	Albrecht model was used because it was able to measure organizational intelligence and for the performance measurement	
